# Linking cell function with perfusion: insights from the transcatheter delivery of bone marrow-derived CD133^+^ cells in ischemic refractory cardiomyopathy trial (RECARDIO)

**DOI:** 10.1186/s13287-018-0969-z

**Published:** 2018-09-14

**Authors:** Beatrice Bassetti, Corrado Carbucicchio, Valentina Catto, Elisa Gambini, Erica Rurali, Alberto Bestetti, Giuseppe Gaipa, Daniela Belotti, Fabrizio Celeste, Matteo Parma, Stefano Righetti, Lorenza Biava, Maurizio Arosio, Alice Bonomi, Piergiuseppe Agostoni, Paolo Scacciatella, Felice Achilli, Giulio Pompilio

**Affiliations:** 1 Vascular Biology and Regenerative Medicine Unit, Centro Cardiologico Monzino-IRCCS, Via Carlo Parea 4, 20138 Milan, Italy; 2 Heart Rhythm Center, Centro Cardiologico Monzino-IRCCS, Via Carlo Parea 4, 20138 Milan, Italy; 3 Service of Nuclear Medicine, IRCCS Multimedica, Via Milanese 300, 20099 Sesto San Giovanni, Milan Italy; 4 Laboratory of Cell and Gene Therapy “Stefano Verri”, ASST-Monza, San Gerardo Hospital, Via Pergolesi 33, 20900 Monza, Italy; 5 Tettamanti Research Center, Tettamanti Foundation, Via Pergolesi 33, 20900 Monza, Italy; 6 University of Milano Bicocca, Via Pergolesi 33, 20900 Monza, Italy; 7 Cardiovascular Imaging Area, Centro Cardiologico Monzino-IRCCS, Via Carlo Parea 4, 20138 Milan, Italy; 8 Haematology Division and BMT Unit, ASST-Monza, San Gerardo Hospital, Via Pergolesi 33, 20900 Monza, Italy; 9 Department of Cardiology, ASST-Monza, San Gerardo Hospital, Via Pergolesi 33, 20900 Monza, Italy; 10 Department of Cardiovascular and Thoracic Diseases, Città della Salute e della Scienza Hospital, Corso Bramante 88, 10126 Turin, Italy; 11 Nuclear Medicine Unit, ASST-Monza, San Gerardo Hospital and University of Milano Bicocca, Via Pergolesi, 33, 20900 Monza, Italy; 12 BioStatistical Unit, Centro Cardiologico Monzino-IRCCS, Via Carlo Parea 4, 20138 Milan, Italy; 13 Heart Failure, Clinical Cardiology and Rehabilitation Cardiology Unit, Centro Cardiologico Monzino-IRCCS, Via Carlo Parea 4, 20138 Milan, Italy; 14 Dipartimento di Scienze Cliniche e di Comunità, Università degli Studi di Milano, Via Festa del Perdono 7, 20122 Milan, Italy

## Abstract

**Background:**

Cell therapy with bone marrow (BM)-derived progenitors has emerged as a promising therapeutic for refractory angina (RA) patients. In the present study, we evaluated the safety and preliminary efficacy of transcatheter delivery of autologous BM-derived advanced therapy medicinal product CD133^+^ cells (ATMP-CD133) in RA patients, correlating perfusion outcome with cell function.

**Methods:**

In the phase I “Endocavitary Injection of Bone Marrow Derived CD133^+^ Cells in Ischemic Refractory Cardiomyopathy” (RECARDIO) trial, a total of 10 patients with left ventricular (LV) dysfunction (ejection fraction ≤ 45%) and evidence of reversible ischemia, as assessed by single-photon emission computed tomography (SPECT), underwent BM aspiration and fluoroscopy-based percutaneous endomyocardial delivery of ATMP-CD133. Patients were evaluated at 6 and 12 months for safety and preliminary efficacy endpoints. ATMP-CD133 samples were used for in vitro correlations.

**Results:**

Patients were treated safely with a mean number of 6.57 ± 3.45 ×  10^6^ ATMP-CD133. At 6-month follow-up, myocardial perfusion at SPECT was significantly ameliorated in terms of changes in summed stress (from 18.2 ± 8.6 to 13.8 ± 7.8, *p* = 0.05) and difference scores (from 12.0 ± 5.3 to 6.1 ± 4.0, *p* = 0.02) and number of segments with inducible ischemia (from 7.3 ± 2.2 to 4.0 ± 2.7, *p* = 0.003). Similarly, Canadian Cardiovascular Society and New York Heart Association classes significantly improved at follow-up vs baseline (*p* ≤ 0.001 and *p* = 0.007, respectively). Changes in summed stress score changes positively correlated with ATMP-CD133 release of proangiogenic cytokines HGF and PDGF-bb (*r* = 0.80, *p* = 0.009 and *r* = 0.77, *p* = 0.01, respectively) and negatively with the proinflammatory cytokines RANTES (*r* = − 0.79, *p* = 0.01) and IL-6 (*r* = − 0.76, *p* = 0.02).

**Conclusion:**

Results of the RECARDIO trial suggested safety and efficacy in terms of clinical and perfusion outcomes in patients with RA and LV dysfunction. The observed link between myocardial perfusion improvements and ATMP-CD133 secretome may represent a proof of concept for further mechanistic investigations.

**Trial registration:**

ClinicalTrials.gov, NCT02059681. Registered 11 February 2014.

## Background

Continual technical improvements in mechanical coronary revascularization have produced as a paradoxical effect a growing number of patients with severe coronary disease, which are no more suitable for further revascularizations and experience refractory angina (RA) despite best medical management. It has been estimated that RA approximates an incidence as high as 50,000 and 30,000–50,000 new cases per year in the USA and continental Europe, respectively [1, 2]. Although the mortality prognosis has improved over time, with current rates approaching 3–5% per year [3, 4], the morbidity counterpart of such a challenging condition still remains a relevant burden for patients as well as health care systems. In particular, RA patients complain of inadequate pain relief leading to revisits to local hospital emergency departments, and undergo repeated coronary investigations [5]. The presence of left ventricular (LV) dysfunction worsens this scenario, contributing to reduce long-term survival and to increase hospitalization rates [2]. In this context, it has been unambiguously shown that ischemia reduction has a pivotal importance for patient survival [6]. Available treatment options on top of best interventional and medical therapy are still limited. Enhanced external counterpulsation, shock waves and coronary sinus reduction [7, 8] have shown therapeutic potential, although none of them has so far gained enough acceptance to enter the routine therapeutic armamentarium.

Cell therapy (CT) with autologous bone marrow (BM)-derived or peripheral blood (PB)-derived vasculogenic cells has emerged as an alternative viable therapeutic option [9, 10]. Randomized controlled trials (RCT) as well as meta-analyses and pooled analyses have recognizably shown, in large cohorts of RA patients, that catheter-based intramyocardial injection of BM or PB-derived selected or unselected progenitors has the ability to improve symptoms, exercise capability and myocardial perfusion with durable effects [11–13].

Nevertheless, in order to gain widespread acceptance and reimbursement from health care systems, pivotal studies as well as large RCT better addressing the cell mode of action (MoA) are still awaited [14]. Moreover, the priority issue about safety and efficacy in the subset of RA patients with LV dysfunction remains to be confirmed, for which less information is available in the literature.

Our group has pioneered the feasibility and safety of direct intramyocardial delivery of BM-derived CD133^+^ progenitors when injected epicardially through a minimally invasive approach in the context of a “no-option” RA population [15]. Furthermore, we validated the good manufacturing practice (GMP)-compliant standard operative procedures (SOP) required to translate human BM-derived CD133^+^ cells as an autologous advanced therapy medicinal product (ATMP-CD133) in the cardiovascular scenario [16, 17].

We here report 1-year safety and preliminary efficacy results of the transcatheter intramyocardial injection of ATMP-CD133 in RA patients with LV dysfunction and concomitant heart failure (HF) and the correlation with myocardial perfusion outcome with CD133^+^ cell function.

## Methods

### Study design

The “Endocavitary Injection of Bone Marrow Derived CD133^+^ Cells in Ischemic Refractory Cardiomyopathy” (RECARDIO) trial is a prospective, multicenter, unblinded, phase I clinical study (ClinicalTrials.gov NCT02059681). The trial was performed at two investigational sites: Centro Cardiologico Monzino-IRCCS (Milan, Italy) as the coordinating and recruiting center, and Città della Salute e della Scienza Hospital (Turin, Italy) as the recruiting center. The institutional review board at each center approved the protocol and all patients gave informed consent prior to participation. Patients were excluded in the case of denial and might withdraw from the study at any time, irrespective of the reason. The study complied with the Declaration of Helsinki and was approved by the local ethical committees (CCFM225/612 and CS/154) and the Italian Competent Authority (Istituto Superiore di Sanità, 15,934(13)PRE21-1199).

**Fig. 1:**
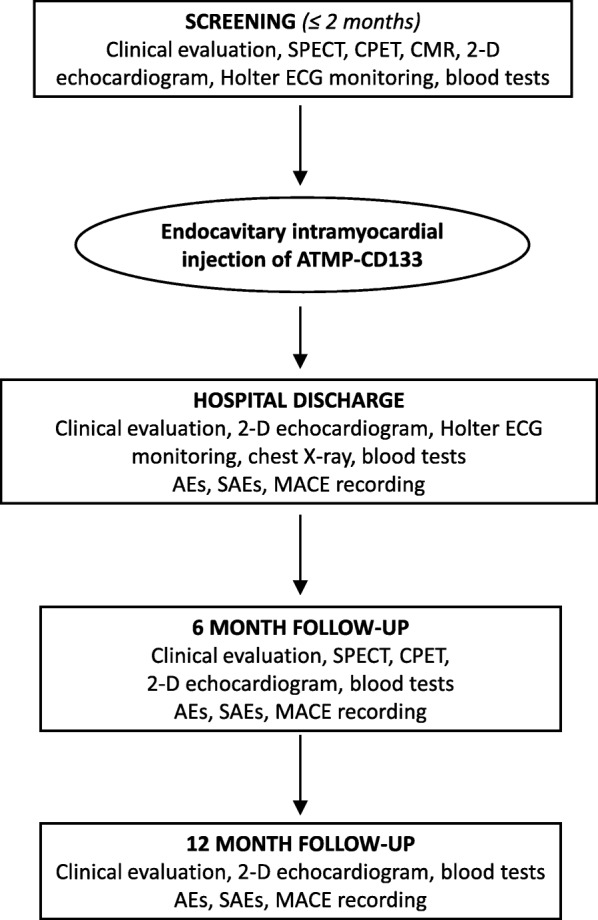
Study flow chart. SPECT gated-single photon emission computed tomography, CPET cardiopulmonary exercise testing, CMR cardiac magnetic resonance, 2D two-dimensional, ECG electrocardiogram, ATMP advanced therapy medicinal product, AE adverse event, SAE serious adverse event, MACE major adverse cardiac events

The study design comprised a screening phase in which patients were checked for inclusion/exclusion criteria and post-procedure follow-up visits scheduled at 6 and 12 months, as safety and efficacy endpoints (Fig. 1). Specifically, the baseline screening assessment was performed within 2 months before the injection procedure and included: clinical evaluation as Canadian Cardiovascular Society (CCS) and New York Heart Association (NYHA) classes, concomitant medications, patients’ quality of life and health status; single photon emission computed tomography (SPECT); cardiopulmonary exercise testing (CPET); two-dimensional (2D) echocardiogram; cardiac magnetic resonance (CMR) when applicable; Holter ECG monitoring; and blood tests. The patients’ quality of life and health status were evaluated using the Short Form-12 (SF-12) survey and the Minnesota Living with Heart Failure Questionnaire (MLHFQ). In particular, the SF-12 survey consists of two parts—the Physical Component Summary (PCS) and the Mental Component Summary (MCS)—while the MLHFQ is a 21-question tool to test patients’ perception of the impact of HF.

At 6 and 12 months, clinical evaluation, 2D echocardiogram and blood tests were repeated. Moreover, SPECT and CPET were also evaluated at 6 months. Adverse events (AE), serious adverse events (SAE) and major adverse cardiac events (MACE) were continuously evaluated up to 12 months.

### Study population

The study population consisted of patients with severe CCS angina class III–IV and/or NYHA score II–IV under maximal tolerated medical therapy for at least 3 months not eligible for any type of conventional mechanical revascularization procedure based on the most recent coronary angiography (≤ 12 months). Additional inclusion criteria were: LV ejection fraction (EF) ≤ 45% as determined by 2D echocardiogram, CMR, or left ventriculogram; a reversible perfusion defect ≥ 10% of the LV surface by SPECT; and peak oxygen consumption (VO_2_) ≤ 21 ml/kg/min at CPET. Major exclusion criteria included: myocardial infarction (MI) within 3 months; presence of LV thrombus; LV wall thickness < 8 mm at the target area of cell injections as assessed by 2D echocardiogram; mechanical aortic valve; severe renal failure (creatinine plasma level > 2.5 mg/dl); positive infectious-disease test for HIV, hepatitis B or C, *Treponema pallidum*, human T-cell lymphotropic virus 1 and 2; and history of malignancy in the past 5 years.

### Study endpoints

The primary aim of the study was safety in terms of any treatment-emergent SAE documented up to 6 months post catheterization and defined as cardiac perforation, pericardial tamponade, sustained ventricular tachycardia/fibrillation and ectopic tissue formation. Additional safety endpoints were evaluated up to 12 months post procedure and included any AE, SAE and MACE defined as composite of death, nonfatal MI, stroke and hospitalization for worsening of HF.

The secondary aim was the efficacy of the procedure in terms of improvements of LV perfusion at SPECT and/or functional capacity as assessed by CPET at 6 months postoperatively.

The third aim was to correlate perfusion and functional benefits with in vitro angiogenic potency of injected cells in terms of endothelial differentiation, colony forming unit capacity and cytokine production.

### Cell preparation

Approximately 350 ml of BM was aspirated from the posterior iliac crest under epidural anesthesia according to the anesthesiologist’s judgment. The procedures were performed by an experienced hematologist within a qualified operating room. BM blood samples were anticoagulated with sodium heparin 5000 UI, stored in bags and shipped at controlled temperature (+ 4 °C/+ 20 °C) to the GMP-qualified facility (Cell and Gene Therapy Laboratory “Stefano Verri”, Monza, Italy).

Since 2007, the Laboratory has been cleared by the Italian Medicines Agency (Agenzia Italiana del Farmaco (AIFA)) for the manufacturing of ATMPs (current authorization aM-185 2017), including ATMP-CD133.

The manufacturing, quality control and transport processes were performed and documented in agreement with validated SOP and GMP requirements.

Each lot was released if the following specifications were met: cellularity 1–12 × 10^6^ CD133^+^ cells; purity (% of CD133^+^ cells/CD45^+^ cells) ≥ 80%; vitality ≥ 80%; endotoxin < 0.5 EU/ml; and sterile. Cells were stored overnight, resuspended in 10 ml of physiological saline, aliquoted in a sterile polypropylene conical tube and packed by the manufacturer as a “ready-to-use” cell product. Based on our stability data [17], cell suspensions were administered within 24 h from the lot release.

### Cell administration and postprocedure monitoring

The day following BM aspiration, patients were admitted to the catheterization laboratory to receive the fluoroscopy-based transendocardial cell injections. The injection technique was performed as previously described [18]. Briefly, selection of target injection sites was done by merging coronary angiography, SPECT and 2D echocardiogram imaging. Upon retrograde LV catheterization, two-view ventriculography was performed to trace LV chamber end-diastolic/end-systolic profiles and color-mark the target landing zone; the different sites for infusion were subsequently chosen within this area. To better guide the Helical Infusion Catheter (BioCardia Inc.), real-time intracardiac echocardiography (ICE) was used in all cases to monitor the appropriate intramyocardial location of the needle during cell delivery and to exclude the release of microbubbles into the cavity. Furthermore, ICE was able to directly visualize the cardiac structures (such as valves and ventricle papillary muscles) and promptly detect procedural complications (such as thrombus formation, valve damage, pericardial effusion, cardiac tamponade). In selected cases, 3D electroanatomical mapping (CARTO; BiosenseWebster) was obtained in the area of interest to validate tissue viability by electrogram analysis at the site of injections [19].

The 10 ml total volume was delivered by means of a range of 11–13 injections by slow infusion of 0.5–0.7 ml from the aliquot syringes over 30 s. At the end of the injection procedure, an implantable loop recorder (ILR) was subcutaneously inserted in the upper chest area to provide 12-month continuous arrhythmia monitoring.

To rule out cardiac tamponade or other cardiac-related complications, all patients underwent a postprocedure 2D echocardiogram and then were monitored overnight by telemetry and measuring vital signs in the intensive care unit.

Patients were hospitalized for a minimum of 48 h after the procedure. Before discharge, 2D echocardiogram, 24-h ECG Holter monitoring, standard chest X-ray imaging and routine blood tests were checked.

### Myocardial perfusion analysis

SPECT perfusion studies were performed at baseline and 6 months after the procedure to evaluate the presence and the extent of reversible ischemia, expressed as a percentage of the LV. Stress and rest SPECT images were acquired 15–60 min after the injection of 555 MBq ^99m^Tc-Tetrofosmin. ECG-SPECT images were performed with patients in the supine position for a total of 64 projections, 3° interval, every 30 s, over a 180° elliptical orbit. Pharmacological stress with intravenous administration of dipyridamole (0.84 mg/kg over 6 min) or regadenoson (0.4 mg/5 ml) was used to obtain stress images.

Both stress and rest acquisitions were obtained using a dual-head digital camera at 90° geometry equipped with high-resolution collimators. LVEF and volumes were calculated using a completely automated algorithm, previously described and validated [20].

The gated images were normalized to the region of highest activity on the end-systolic image set. Regional myocardial perfusion was assessed on the summed images. Perfusion imaging was scored semi-quantitatively using a 20-segment model scored on a 5-point scale (0 = normal, 4 = no uptake) [21]. A perfusion defect with a score ≥ 3 was considered significant, and a segment score improved by at least one grade was considered reversible. The summed stress score (SSS) and summed rest score (SRS) were calculated in each patient by adding the 20 individual perfusion scores. The difference between the summed stress and rest scores is the summed difference score (SDS), which indicated the amount and the degree of reversible ischemia. The paired SPECT images were evaluated by two independent imaging readers blinded to the clinical data.

### Echocardiographic and functional capacity assessments

A 2D echocardiogram was performed at baseline, before hospital discharge and after 6 and 12 months to evaluate LV function, volumes and wall motion score index (WMSI). Pericardial effusions and unwanted tissue changes were also assessed during the study period. Echocardiogram acquisition and analyses were performed according to the guidelines [22]. The 2D echocardiogram at screening was also applied to assess the presence and localization of LV wall thickness < 8 mm at the target sites of cell injections.

CPET studies were performed at baseline and 6 months postoperatively to assess changes in functional capacity. CPET were performed on a cycle ergometer using an incremental ramp protocol. Expiratory O_2_, CO_2_ and ventilation were measured breath by breath. Patients were encouraged to perform as maximal exercise as possible. Peak VO_2_ was reported as a mean over the last 20 s of exercise.

### ATMP-CD133 in vitro analyses

**Fig. 2:**
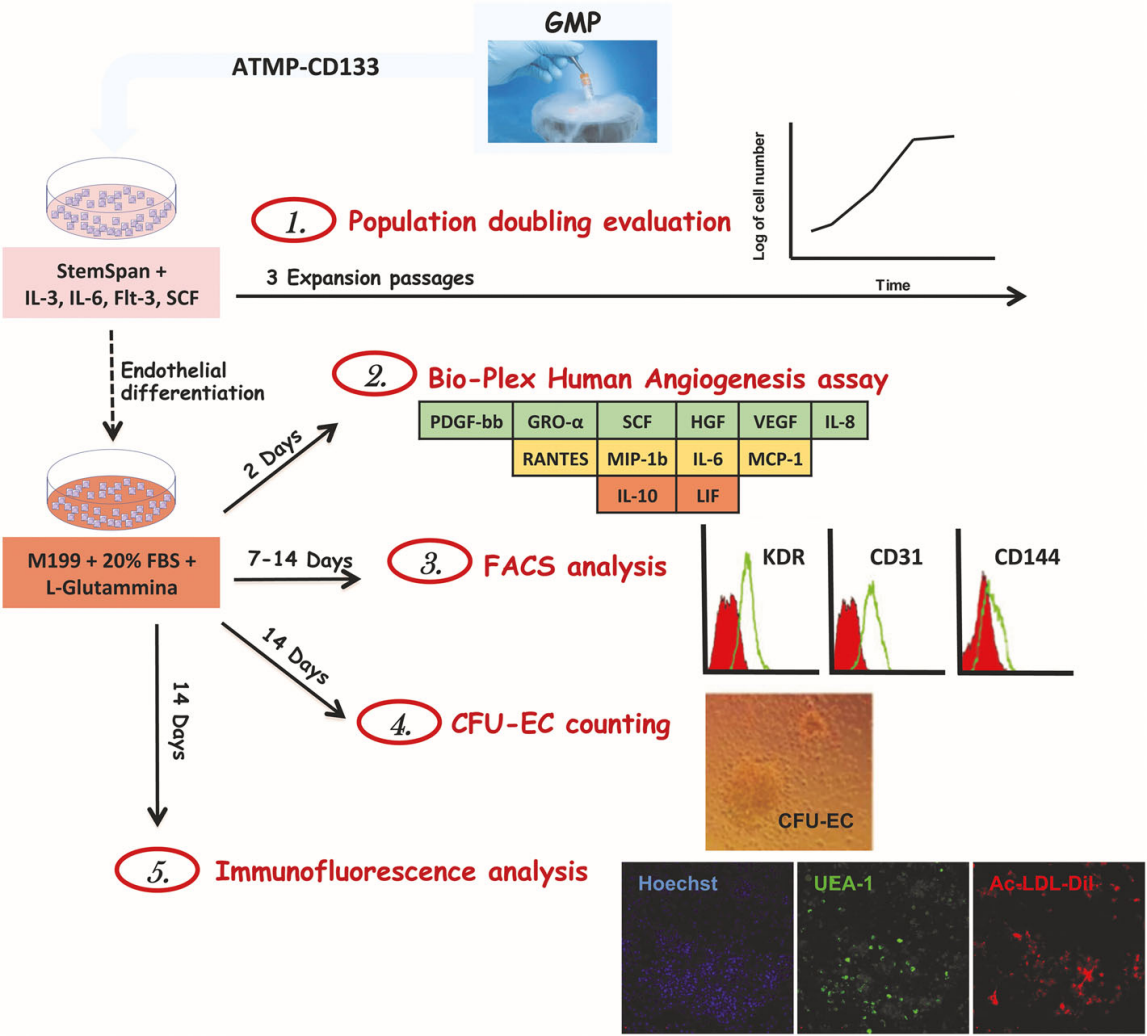
Schematic representation of the in vitro experimental plan. GMP good manufacturing practice, ATMP advanced therapy medicinal product, IL interleukin, SCF stem cell factor, FBS fetal bovine serum, PDGF-bb platelet-derived growth factor type bb, GRO-α growth-regulated oncogene alpha, HGF hepatocyte growth factor, VEGF vascular endothelial growth factor, RANTES regulated on activation normal T cell expressed and secreted, MIP-1b macrophage inflammatory protein-1 beta, MCP-1 monocyte chemoattractant protein-1, LIF leukemia inhibitory factor, FACS fluorescence-activated cell sorting, CFU-EC colony forming unit-endothelial cell, Ac-LDL-Dil acetylated low-density lipoprotein labeled with dioctadecyl-tetramethylindocarbocyanine perchlorate, UEA-1 *Ulex europaeus* agglutinin-1

A small amount of each ATMP-CD133 lot was obtained from the GMP facility to perform in vitro studies. The experimental plan is shown in Fig. 2.

In detail, samples were thawed and seeded at 10^5^ cells/well in 96-well plates in StemSpan (STEMCELL Technologies) supplemented with interleukin (IL)-3 and IL-6 (both at 20 ng/ml; Peprotech), flt3 ligand (FLT3LG) and stem cell factor (SCF) (both at 100 ng/ml; Peprotech) to allow cell proliferation. The ATMP-CD133 growing capacity was assessed using the cumulative population doubling levels (CPDL), as previously described [23].

After three expansion passages, samples were seeded onto Fibronectin (Sigma-Aldrich)-coated dishes in M199 medium (Gibco) supplemented with 20% fetal bovine serum (FBS; Microtech), 2 mM l-glutamine (Euroclone) and 100 U/ml penicillin/streptomycin. Seeded cells were cultured for 2, 7 or 14 days to carry out the secretome and the flow cytometry analyses, to measure the production of colony forming unit-endothelial cells (CFU-EC) and to assess the immunophenotype of cultured cells. In particular, after 2 days, ATMP-CD133 secretome (expressed as pg/ml/10^5^ cells) was characterized using a customized Bio-Plex assay (BIO-RAD). The panel comprised six proangiogenic factors including SCF, growth-regulated oncogene alpha (GRO-α), vascular endothelial growth factor (VEGF), platelet-derived growth factor type bb (PDGF-bb), hepatocyte growth factor (HGF) and IL-8; four proinflammatory factors including monocyte chemoattractant protein-1 (MCP-1), macrophage inflammatory protein-1 beta (MIP-1β), regulated on activation normal T cell expressed and secreted (RANTES) and IL-6; and two anti-angiogenic factors including leukemia inhibitory factor (LIF) and IL-10. As a negative control, nonconditioned medium was tested.

Immunophenotype analysis of endothelial markers (CD31, KDR, CD144) [24] was performed by multicolor flow cytometry on cultured cells after 7 and 14 days of endothelial conditioning. After detachment, using a nonenzymatic method, cells were resuspended in washing buffer (WB) containing PBS, 0.1% BSA (Gibco) and 2 mM EDTA (Gibco), and incubated in the dark for 15 min with suitable combinations of the following monoclonal or isotype-matched control antibodies: CD31-FITC (clone WM59; BD), KDR-PE (clone 89,106; R&D Systems) and CD144-APC (clone 16B1; R&D Systems). Then, samples were washed with 1 ml of WB and centrifuged for 10 min at 400 × *g* at 4 °C to remove unbound antibodies. Cells were then resuspended in 250 μl of WB and analyzed with a Gallios™ Flow Cytometer (Beckman Coulter).

After 14 days in differentiation-promoting conditions, a CFU-EC assay was performed as previously described [16]. For immunofluorescence analysis, cells were incubated in the dark for 5 h at 37 °C with 10 μg/ml of acetylated low-density lipoprotein labeled with dioctadecyl-tetramethylindocarbocyanine perchlorate (Ac-LDL-Dil; Biomedical Technologies). After washing with PBS, cells were fixed with 4% paraformaldehyde (Sigma-Aldrich) for 20 min and then stained with 40 μg/ml of FITC-labeled Lectin from *Ulex europaeus* agglutinin-1 (UEA-1 Lectin; Sigma-Aldrich) in the dark for 1 h. Nuclei were stained with Hoechst 333,428 (Sigma-Aldrich) in the dark for 15 min. Cells were observed with a Zeiss LSM 710 confocal microscope.

### Statistical analyses

Continuous variables were expressed as mean ± SD or median (interquartile range (IQR)), as appropriate. A within-subject Student’s *t* test was used to compare baseline and 6-month follow-up data. To evaluate differences in the distribution of continuous data at baseline, 6-month and 12-month follow-up, one-way ANOVA or the Friedman test for repeated measures were performed with Bonferroni or Dunn’s post-hoc analysis, respectively. Correlations between continuous variables were assessed by Pearson or Spearman test, as appropriate.

All tests were two-tailed, with a statistically significant *p* ≤ 0.05. All of the analyses were performed with GraphPad Prism® software (version 5.0).

## Results

### Patient characteristics

Between December 2013 and November 2016, 10 consecutive patients were enrolled and followed up for a period of 12 months according to the study protocol. Baseline characteristics are presented in Table 1. All patients were males and the mean age was 69.4 ± 3.8 years. All patients had a history of coronary artery bypass grafting and seven patients experienced MI. Two patients were implantable cardioverter defibrillator (ICD) recipients and two patients had a spinal cord stimulator. Medications at baseline, including the use of long-lasting nitroglycerin and ranolazine to manage RA, are presented in Table 1.

**Table 1:** Patients’ characteristics

Characteristic	Baseline
Age (years), mean ± SD	69.4 ± 3.8
Males, *n*/total	10/10
BMI (kg/m^2^), mean ± SD	26.3 ± 2.4
Cardiovascular risk factors, *n*/total	
Current smoking	7/10
Diabetes mellitus	6/10
Hypertension	10/10
Hypercholesterolemia	9/10
Family history of CAD	6/10
Medical history, *n*/total	
Prior CABG	10/10
Prior MI	7/10
Prior PCI	8/10
Prior ICD implant	2/10
Spinal cord stimulation	2/10
Peripheral vascular disease	1/10
Current medications, *n*/total	
β-Blockers	9/10
ACE inhibitors	5/10
ARBs	3/10
Calcium antagonists	1/10
Diuretics	6/10
Statins	10/10
Aspirin	8/10
Ranolazine	7/10
Long-lasting nitroglycerin	6/10
Cardiovascular condition at enrolment, mean ± SD	
LVEF (%)	38.3 ± 5.1
LV reversible ischemia (%)	15.3 ± 8.4
Peak VO_2_ (ml/kg/min)	13.3 ± 1.8

Continuous data presented as mean ± SD

*SD* standard deviation, *BMI* body mass index, *CAD* coronary artery disease, *CABG* coronary artery bypass grafting, *MI* myocardial infarction, *PCI* percutaneous coronary intervention, *ICD* implantable cardioverter defibrillator, *ACE* angiotensin converting enzyme, *ARB* angiotensin II receptor blocker, *LVEF* left ventricular ejection fraction, *LV* left ventricle, *VO*_*2*_ oxygen consumption

### BM harvesting and ATMP-CD133 lot release

A mean 349 ± 57 ml of BM was aspirated under epidural anesthesia in the absence of adverse events. The mean procedural time was 24 ± 10 min.

At the end of the GMP manufacturing process, the mean number of ATMP-CD133 was 6.57 ± 3.45 × 10^6^ cells. The median (IQR) cell purity and vitality was 88.77% (86.30–90.75%) and 99.90% (99.85–99.95%), respectively (Table 2).

**Table 2:** ATMP-CD133 production and delivery

Parameter	Value
BM harvest (ml)	349 ± 57
ATMP-CD133 production	
Cellularity (× 10^6^)	6.57 ± 3.45
Purity (%)	88.77 (86.30–90.75)
Vitality (%)	99.90 (99.85–99.95)
ATMP-CD133 delivery	
Number of injections	12 (11–12)
Procedural duration (min)	252 ± 91
Mapping and injection time (min)	105 ± 29
Fluoroscopy time (min)	63 ± 22

Data presented as mean ± standard deviation or median (interquartile range), as appropriate

*ATMP* advanced therapy medicinal product, *BM* bone marrow

### Transendocardial delivery of ATMP-CD133

Fluoroscopy-guided endocavitary intramyocardial cell injections were successfully accomplished in all 10 patients. Patients received a mean total volume of 7.76 ± 0.8 ml, delivered in a median of 12 independent injections of 0.5–0.7 ml each. All injections were double-checked with ICE to confirm engagement of the needle into the LV wall. Mean total procedural duration was 252 ± 91 min, while the mapping and injection time averaged 105 ± 29 min (Table 2). In four cases, the injection procedure was preceded by CARTO-guided electroanatomical mapping. An ILR was implanted in eight patients, with the exception of two cases who were already recipients of an ICD.

### Safety profile of ATMP-CD133

There were no treatment-emergent in-hospital SAE related to the transendocardial delivery of ATMP-CD133. In 3 out of 10 patients, mild to moderate (≤ 15 mm) pericardial effusions were recorded on 2D echocardiogram during the 24 h postoperative monitoring. These events were all spontaneously resolved before hospital discharge. During the 12-month arrhythmia follow-up, no patient presented episodes of sustained ventricular tachycardia or ventricular fibrillation as documented by ILR or ICD continuous monitoring.

No patient died during the 1-year safety follow-up. Two MACE and six nonprocedure-related SAE occurred during the 12-month study period. As for MACE, two patients experienced nonfatal non-ST elevation MI at 8 and 12 months, respectively, after the injection procedure. The first patient received PCI of the right coronary artery in territories distal from the targeted injection area and the second was managed conservatively based on coronary angiography. Two patients were treated with ICD implantation in primary prevention 6 months after the cell treatment. One patient reported two SAE during the follow-up, requiring emergency room admission for epigastric ulcer and subdural hematoma after 2 and 8 months, respectively. Finally, one patient, with known peripheral vascular disease, underwent elective percutaneous angioplasty of the femoral superficial artery 5 months after the procedure.

### Perfusion outcome

**Fig. 3:**
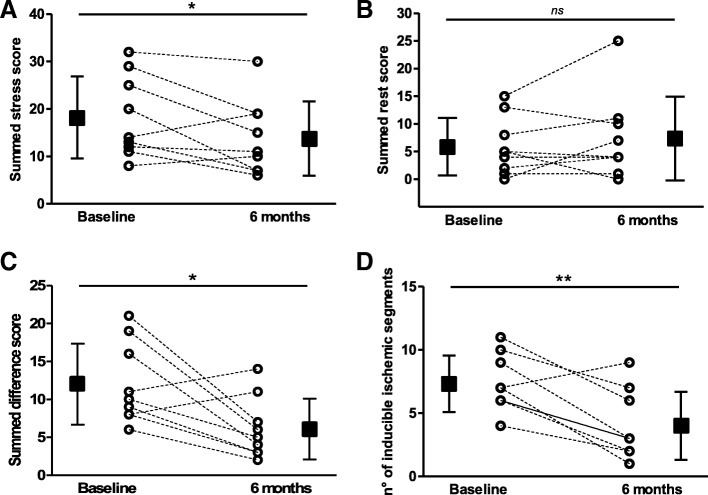
Myocardial perfusion at SPECT after 6-month follow-up. Changes of **a** summed stress score, **b** summed rest score, **c** summed difference score and **d** number of segments with inducible ischemia per patient. Square data markers with error bars represent mean ± SD of each SPECT parameter at baseline and 6 months (*n* = 9). *p ≤ 0.05, **p ≤ 0.01, ns = not significant

Follow-up SPECT was available in 9 out of 10 patients. Myocardial perfusion was significantly ameliorated at 6 months compared with baseline. In particular, the SSS improved from 18.2 ± 8.6 to 13.8 ± 7.8 (*p* = 0.05; Fig. 3a) while the SRS did not change significantly (5.9 ± 5.2 vs 7.3 ± 7.9, *p* = 0.38; Fig. 3b). Moreover, the SDS decreased from 12.0 ± 5.3 to 6.1 ± 4.0 (*p* = 0.02; Fig. 3c). As for ischemic myocardial segments, a highly significant difference was found in the number of segments showing inducible myocardial ischemia (from 7.3 ± 2.2 to 4.0 ± 2.7, *p* = 0.003; Fig. 3d).

### Echocardiographic and functional outcome

LVEF, LV volumes and WMSI did not change significantly during the follow-up, as assessed by 2D echocardiogram (Table 3).

**Table 3:** Echocardiographic findings at baseline and after 6 and 12 months

Parameter	Baseline	6 months	12 months	*p* value
LVEF (%)	41.1 ± 9.5	40.9 ± 11.0	39.5 ± 10.7	0.76
LVESV (ml)	93.4 ± 38.5	92.1 ± 36.6	96.3 ± 40.1	0.78
LVEDV (ml)	153.5 ± 45.0	152.1 ± 33.7	154.2 ± 36.9	0.96
WMSI	1.8 ± 0.5	1.8 ± 0.5	1.9 ± 0.5	0.43

Data presented as mean ± standard deviation

*LVEF* left ventricular ejection fraction, *LVESV* left ventricular end-systolic volume, *LVEDV* left ventricular end-diastolic volume, *WMSI* wall motion score index

Similarly, no differences were recorded between baseline and 6 months in peak VO_2_ on CPET (13.3 ± 1.8 vs 14.1 ± 3.3, *p* = 0.37).

### Clinical outcome

**Fig. 4:**
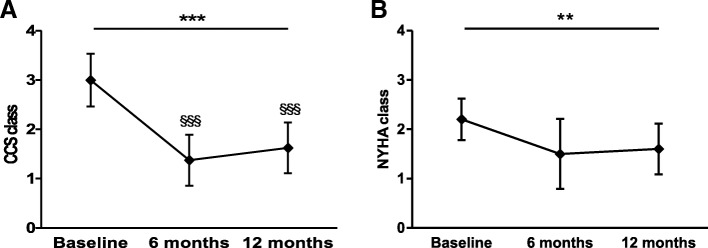
Canadian Cardiovascular Society and New York Heart Association classes over 12-month follow-up. **a** Canadian Cardiovascular Society (CCS) class changes from 6 and 12 months to baseline (*n* = 8). **b** New York Heart Association (NYHA) class changes from 6 and 12 months to baseline (*n* = 10). Data presented as mean ± SD. **p ≤ 0.01, ***p ≤ 0.001. ^§§§^*p* ≤ 0.001 vs baseline

Patients’ clinical status was assessed according to CCS and NYHA classes, nitrate consumption and disease-related questionnaires at baseline and 6 and 12 months post procedure. Of importance, a significant improvement in the CCS angina class was noted during the follow-up period (*p* ≤ 0.001) in 8 out of 10 patients. In two diabetic patients, no angina symptoms were present at accrual. In particular, the CCS class was observed to be markedly reduced from 3.0 ± 0.5 at baseline to 1.4 ± 0.5 at 6 months (*p* ≤ 0.001) and to 1.6 ± 0.5 at 12 months (*p* ≤ 0.001) (Fig. 4a). Notably, the patient percentage showing a ≥ 2 class improvement was 50% at 6 months and 37.5% at 12 months. In parallel, the NYHA class significantly improved during the 12-month study period (*n* = 10, *p* = 0.007; Fig. 4b). Moreover, the nitrate consumption per week progressively decreased during the study period from 4 (2–7) to 0.25 (0.04–4) at 6 months and 0.04 (0–2) at 12 months (*n* = 7, *p* < 0.0001), with a significant difference in nitrate consumption at 12 months with respect to baseline (*p* ≤ 0.001).

In line with these results, quality-of-life perception suggested trends toward symptomatic benefit. The SF-12 survey showed an improvement of the PCS throughout the follow-up period (*p* = 0.02) with a significant difference in the PCS at 12 months with respect to baseline (*p* ≤ 0.05). Both SF-12 MCS and MLHFQ total score did not change significantly during the follow-up period (Table 4).

**Table 4:** Patients’ quality of life and health status

Parameter	Baseline	6 months	12 months	*p* value
SF-12				
PCS	38.4 ± 4.6	44.0 ± 6.7	44.4 ± 6.0*	0.02
MCS	49.8 ± 9.9	49.5 ± 13.2	49.2 ± 15.2	0.99
MLHFQ	35.9 ± 12.2	27.1 ± 10.7	33.1 ± 18.6	0.29

Data presented as mean ± standard deviation

*SF-12*, Short Form-12, *PCS* Physical Component Summary, *MCS* Mental Component Summary, *MLHFQ* Minnesota Living with Heart Failure Questionnaire

*p ≤ 0.05 vs baseline

### ATMP-CD133 functional profile

**Fig. 5:**
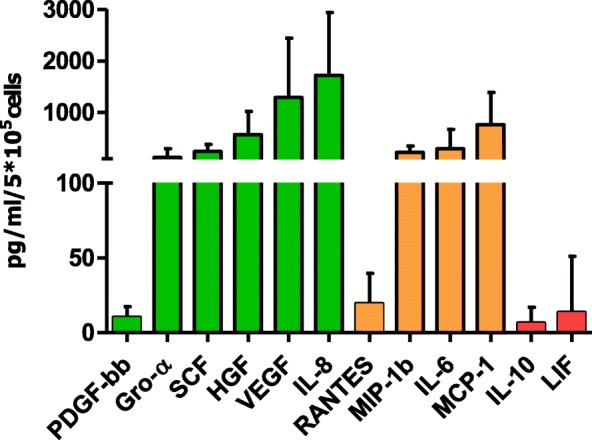
Selected secreted signature of ATMP-CD133. Pro-angiogenic (green bars), pro-inflammatory (orange bars) and anti-angiogenic (red bars) factor expression levels detected in supernatants of ATMP-CD133 (*n* = 10). PDGF-bb platelet derived-growth factor type bb, GRO-α growth-regulated oncogene alpha, SCF stem cell factor, HGF hepatocyte growth factor, VEGF vascular endothelial growth factor, IL interleukin, RANTES regulated on activation normal T cell expressed and secreted, MIP-1b macrophage inflammatory protein-1 beta, MCP-1 monocyte chemoattractant protein-1, LIF leukemia inhibitory factor

ATMP-CD133 samples were obtained in all patients. ATMP-CD133 CPDL derived after three expansion passages varied highly among samples, ranging from 1.45 to 5.59 with a mean value of 3.32 ± 1.33. After a conditioned period of 48 h, ATMP-CD133 supernatants showed the modulation of the expression levels of several proangiogenic, proinflammatory and anti-angiogenic factors (Fig. 5). Among those tested, a large amount of proangiogenic cytokines and growth factors such as IL-8 and VEGF (1722 ± 1218 and 1293 ± 1153 pg/ml/10^5^ cells, respectively) and, in less proportion, proinflammatory cytokines such as MCP-1 (768 ± 619 pg/ml/10^5^ cells) were secreted. Consistently, low levels of anti-angiogenic cytokines, like LIF and IL-10, were found (13.61 ± 37.42 and 6.61 ± 10.42 pg/ml/10^5^ cells, respectively).

Flow cytometry analysis at 7 days showed the expression at high levels of early endothelial markers such as CD31 (35.95 ± 16.55%) and, to a lesser extent, KDR (26.42 ± 28.13%). The expression of CD144, recognized as a terminally differentiated endothelial marker, was detectable in a minority of cells (15.58 ± 18.01%). After 14 days, CD31 and KDR expression levels decreased to 17.95 ± 18.96% and 19.71 ± 18.32%, respectively, while CD144 expression was maintained (14.04 ± 15.99%).

**Fig. 6:**
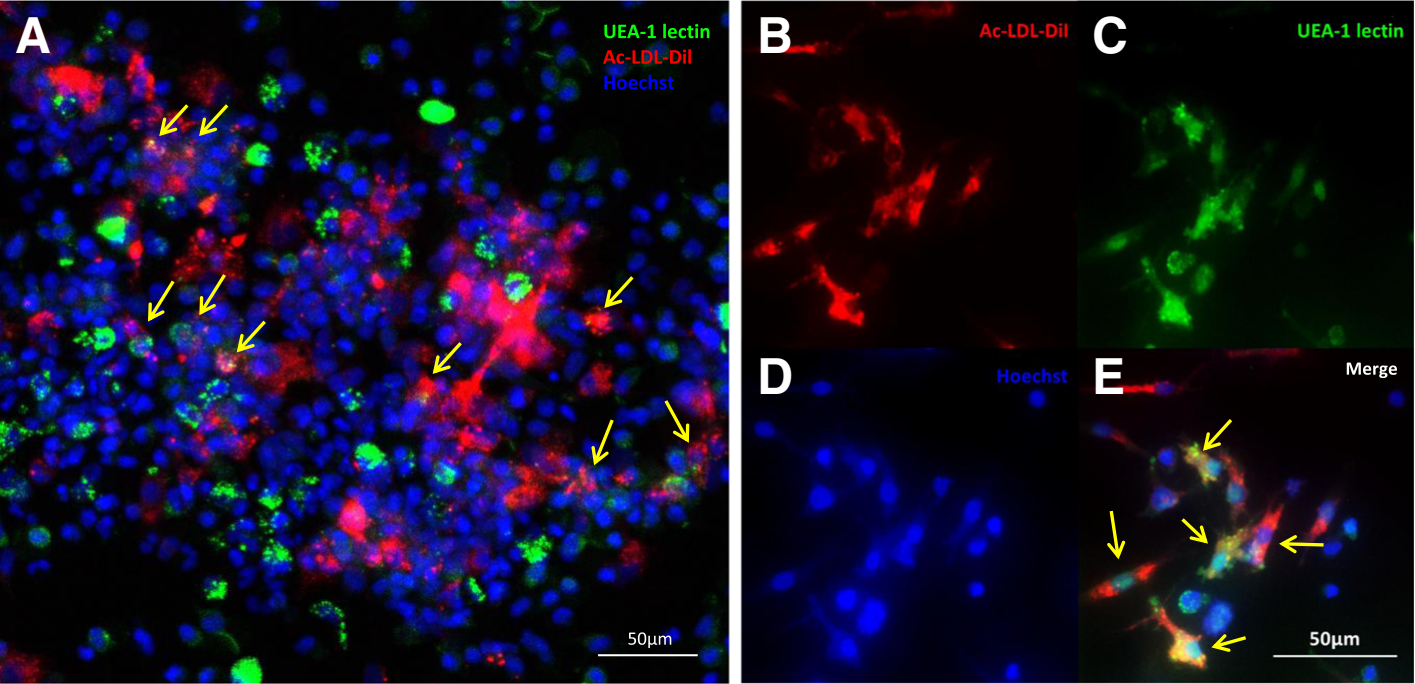
ATMP-CD133 in vitro endothelial differentiation. Representative immunofluorescence of **a** clustered and (**b–e**) not clustered CFU-EC derived from ATMP-CD133 after 14 days in culture. Arrows indicate ATMP-CD133 committed to endothelial lineage, double-positive for Ac-LDL-Dil (red) and UEA-1 lectin (green). UEA-1 *Ulex europaeus* agglutinin-1, Ac-LDL-Dil acetylated low-density lipoprotein labeled with dioctadecyl-tetramethylindocarbocyanine perchlorate

After 14 days under differentiation conditions, it was found that ATMP-CD133 form a number of CFU-EC which was strongly different among samples, ranging from 47 to 700 colonies/10^5^ cells with a median of 150 (85–415). At immunofluorescence, both clustered and nonclustered cells derived from ATMP-CD133 were double-positive for Ac-LDL-Dil and UEA-1 lectin staining, confirming their endothelial commitment (Fig. 6a–e).

### Correlation of ATMP-CD133 secretome with perfusion

**Fig. 7:**
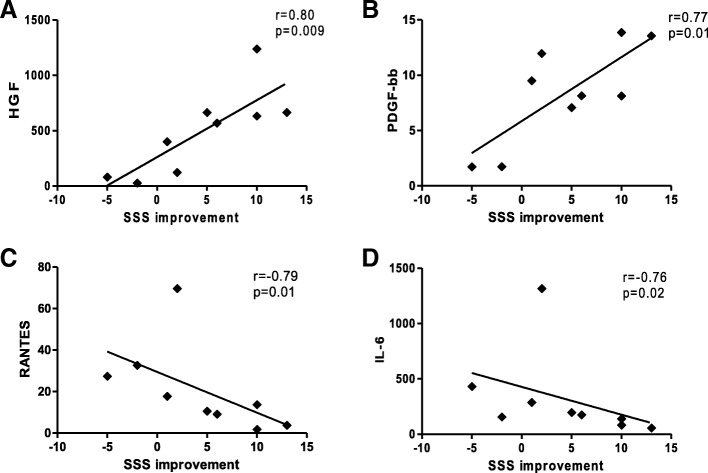
Correlation between ATMP-CD133 secreted factors and myocardial perfusion changes at SPECT. SSS improvements correlate (**a**, **b**) positively with measured levels of proangiogenic factors and (**c**, **d**) negatively with proinflammatory factors (*n* = 9). SSS summed stress score, HGF hepatocyte growth factor, PDGF-bb platelet derived-growth factor type bb, RANTES regulated on activation normal T cell expressed and secreted, IL interleukin

To evaluate whether the ATMP-CD133 functional profile correlates with the observed improvements in myocardial perfusion at SPECT, linear regression analyses were performed. Notably, SSS changes were found to be significantly correlated with the secreted proangiogenic growth factors HGF (*r* = 0.80, *p* = 0.009) and PDGF-bb (*r* = 0.77, *p* = 0.01; Fig. 7a, b). On the contrary, a negative significant correlation with SSS improvements was found for the proinflammatory cytokines RANTES (*r* = − 0.79, *p* = 0.01) and IL-6 (*r* = − 0.76, *p* = 0.02), as shown in Fig. 7c, d. No other correlations have been detected between myocardial perfusion changes and ATMP-CD133 functional profile.

## Discussion

The RECARDIO phase I trial was designed to evaluate safety and preliminary efficacy of transcatheter intramyocardial injections of ATMP-CD133 in patients with RA and LV dysfunction (EF ≤ 45%). The major finding of this pilot study was that LV dysfunction in highly symptomatic RA patients does not alter the positive safety profile of catheter-based intramyocardial cell delivery. Further, a favorable preliminary efficacy signal in terms of SSS and SDS improvements at SPECT between baseline and 6-month follow-up was observed. Finally, a correlation was found for the first time in the RA context between myocardial perfusion changes and the ATMP-CD133 secretome profile.

Refractory angina is a debilitating condition representing an emerging burden for health systems [2]. A recent Ontario-based study conservatively estimated the annualized cost of angina-related disability including direct, indirect and system costs at $19,209 per patient/year [1]. In this scenario, the only pharmacological therapy that has been approved for RA in the last 40 years is the late sodium current blocker ranolazine, whose effectiveness has, however, been recently questioned [25].

Of importance, CT by means of catheter-based intramyocardial cell delivery has emerged as a viable and promising therapy. Different BM or PB-derived autologous vasculogenic cell populations, including unfractioned mononuclear cells [26, 27] and positively selected CD34 [28] or CD133 [29, 30] cells, have been injected into ischemic areas to ameliorate perfusion of LV territories not otherwise amenable to revascularization. Large meta-analyses have concordantly suggested that CT has an overall favorable effect in symptom relief and exercise capacity improvement in “no option” patients with RA [12, 31, 32]. Moreover, a decreased incidence of MACE and arrhythmias in cell-treated patients has been observed [12]. Of note, the transcatheter intramyocardial approach appears to compare favorably to intracoronary infusion in patients with ischemic cardiomyopathy and LV dysfunction [33]. Very recently, a large patient-level pooled analysis of RCT including 304 RA patients [13] has demonstrated consistent and durable improvements in exercise capacity, angina frequency and mortality after intramyocardial injection of autologous CD34^+^ cells.

We and others have described CD133^+^ cells as an immature population of endothelial progenitors highly overlapping with CD34^+^ cells and having enhanced paracrine-driven proangiogenic effects [16, 34–36]. In 2015, we were the first to report the full-GMP compliant validation of BM-derived human CD133^+^ cells to be used as an ATMP for cardiac CT [17] in compliance with the European Medicine Agency (EMA) guidelines [37] and the Committee of Advance Therapies (CAT) recommendations [38].

In the PROGENITOR trial (Selected CD133^+^ Progenitor Cells to Promote Angiogenesis in Patients With Refractory Angina) [29], Jimenez-Quevedo et al. were the first to attempt NOGA-guided endocavitary intramyocardial injection of CD133^+^ cells mobilized from PB in RA patients. Although differences between treated and control groups were not detectable, authors were able to show a significant improvement in CCS class and in the number of angina episodes per month in the treatment arm only. Besides, at 6 months, the summed score improved significantly at rest and stress in the cell arm. On the contrary, in the more recent placebo-controlled REGENT-VSEL study (Transendocardial Delivery of Bone Marrow–Derived CD133^+^ Cells on Left Ventricle Perfusion and Function in Patients With Refractory Angina) [30], Wojakowski et al. did not report any significant reduction of myocardial ischemia and angina vs placebo after NOGA-guided CD133^+^ cell intramyocardial injection, although a mild but significant reduction of LV end-systolic and end-diastolic volumes was observed in the cell group only. Moreover, the odds ratio adjusted for confounding factors showed a 3.5-fold higher positive response at SPECT in patients allocated to the active arm vs placebo. In our RECARDIO pilot trial, the main efficacy signal was a significant intragroup improvement in perfusion at SPECT in terms of the SSS and SDS along with a reduction in the number of segments showing inducible myocardial ischemia after 6 months. Moreover, a consistent amelioration in the CCS and NYHA classes was also appreciated during the 12-month follow-up period.

Several differences have to be taken into account when interpreting such mixed results. Although both the PROGENITOR and REGENT-VSEL trials randomized RA patients vs controls/placebo, whereas the RECARDIO trial did not, these studies were underpowered to detect perfusion differences. However, additional variables may also play a major role. One of the most relevant is probably the level of the ischemic burden. Rodrigo et al. [39] identified the large number of myocardial segments at baseline as an independent predictor of a significant improvement in perfusion, and Wojakowski et al. [30] recognized that the inclusion of patients with a low number of baseline ischemic segments might have influenced their results (3.6 ± 2.7 in the REGENT-VSEL trial vs 7.3 ± 2.2 in the RECARDIO trial). Along the same line, it is likely that the higher angina class, the better the likelihood of improvement, as reflected by the nearly identical and significant 6-month reduction of CCS class that the RECARDIO and PROGENITOR studies have observed, having included patients with mean CCS ≥ 3 at baseline. It is worth mentioning that our findings of a remarkable sustained angina benefit are perfectly in line with a consistent body of previous RCT and meta-analyses confirming clinical efficacy of BM or PB intramyocardial progenitor cell injection in RA patients [13, 40, 41]. One can expect that perfusion and symptom changes may correlate with LV and patients’ functional amelioration. However, this correlation was not evenly observed in previous studies [42, 43], probably reflecting the heterogeneous nature of this patient population.

Other factors, including guidance of cell delivery and the number of injected cells, may also be taken into account to explain variability among studies. Different from the REGENT-VSEL and PROGENITOR studies, in the RECARDIO trial intramyocardial injection was fluoroscopy based rather than NOGA based. Given the difficulty of anatomical matching between injections and SPECT target areas [30], the fluoroscopy-based approach may be postulated as less accurate in targeting injections in viable ischemic areas. This limitation was partially overcome by using electroanatomical mapping to reveal the electrical properties within the area of interest and exclude segments of scar. Besides, the routine use of ICE contributed to monitoring the needle engagement into the myocardial wall [19]. Overall, in our experience, the fluoroscopy-based approach has confirmed feasibility, safety and practicality as previously reported in larger studies [44, 45]. Moreover, the unique design of the Helical Infusion Catheter has been recently proved to provide a cell retention 3-fold higher than a straight needle approach [46].

As for the cell number, even if no dose-finding studies are available for CD133^+^ cells in RA, we have delivered into the myocardium a mean of 6.5 × 10^6^ CD133^+^ cells, a cell dosage 2-fold higher than the REGENT-VSEL study, which is the only one of the RCT in RA delivering selected CD133^+^ cells obtained from the BM.

### Safety aspects

The specificity of our study with respect to previous ones in RA was the inclusion of patients with moderate to severe LV dysfunction (baseline EF 38.3%), a scenario that may worsen the safety of CT. Nevertheless, we have confirmed the excellent safety profile of catheter-based intramyocardial cell injection, including the absence of any treatment-emergent SAE. Our results are in line with those reported in the FOCUS-CCTRN trial in which 92 patients with chronic ischemic cardiomyopathy were randomized to transendocardial cell vs placebo injection, without safety issues in terms of mortality and SAE at early and mid-term [47]. In particular, in our study, ILR continuous monitoring confirmed the absence up to 12 months of malignant arrhythmias. Since one of the major safety concerns of transendocardial cell delivery is cardiac tamponade due to perforation, we have routinely utilized ICE to check the real-time status of the pericardium during injections. Such an adjuvant imaging technique was not previously reported in CT studies. Moreover, the long steerable shuttle sheet (Morph) of the Helix delivery technology (BioCardia Inc.) is helpful for safely handling the injection catheter both in the case of peripheral vessel tortuosity and for crossing the aortic valve in the case of calcified cusps.

### Correlations between perfusion and ATMP-CD133 secretome

In the global position paper of the TACTICS group [48], a recommendation has been made to incorporate CT mechanistic endpoints into newly designed clinical trials to corroborate unanswered hypotheses on the MoA. Since a consistent body of evidence suggests paracrine activity of angiogenic cells as the principal MoA [49], there is the need to investigate the link between cell potency and function for ATMP-CD133. To our knowledge, no such previous information is available in the RA context. Our findings correlate for the first time the ATMP-CD133 secretome profile with SSS changes, a recognized perfusion index impacting cardiac prognosis [50]. Specifically, a positive correlation with SSS improvements was found with the proangiogenic growth factors HGF and PDGF-bb, whereas the proinflammatory cytokines RANTES and IL-6 correlate negatively. Interestingly, cardiotrophic growth factors, such as HGF and PDGF-bb, have been consistently described as the main components of the paracrine MoA of BM-derived endothelial progenitors concurring to cooperative neovasculogenesis and exerting chemoattractants as well as stimulatory effects on endothelial and muscle cell growth [51–54]. Conversely and notably, RANTES has been described as a marker of refractory symptoms in plasma of patients with unstable angina [55]. Moreover, proinflammatory chemokines, as IL-6 and RANTES, have been shown to play detrimental inflammatory direct effects on the myocardium [49] as well as to impair survival and differentiation of transplanted endothelial progenitors in the ischemic heart substrate [56]. In our study, we found no correlation with other well-known cytokines related to angiogenesis and inflammation, such as VEGF, and neither we were able to dissect the exact contribution of proinflammatory and anti-inflammatory factors (i.e., IL-6 or MCP-1) to produce the angiogenic effect. Further analyses are required on a larger sample size for a comprehensive assessment of the biological background of cell potency. Nevertheless, we can speculate that our data support the rationale for linking up a cell-specific secretion signature with a potential therapeutic benefit.

#### Study limitations

The RECARDIO trial is a phase I uncontrolled study, so no efficacy claims can be extrapolated. Perfusion improvements detected at SPECT must be confirmed by larger RCT. Another possible study limitation is that the ATMP-CD133 cytokine profile has been gathered from in vitro experiments, which may not fully mimic the in vivo environment. Moreover, correlations observed between cell secretome and SSS improvement have to be only considered as a proof of concept of a possible ATMP-CD133 MoA that needs further mechanistic investigations.

## Conclusions

The RECARDIO trial was the first designed to address safety and preliminary efficacy of intramyocardial injection of ATMP-CD133 in the RA patient subset with LV dysfunction, and to correlate perfusion changes with CD133^+^ cell function. The satisfactory safety profile along with perfusion amelioration at SPECT may open larger controlled investigations in this context. Moreover, the observed link between cell function and SPECT changes hints for the first time in patients at mechanistic insights for a vasculogenic CD133^+^ cell MoA.

## Abbreviations

2D: Two-dimensional
Ac-LDL-Dil: Acetylated low-density lipoprotein labeled with dioctadecyl-tetramethylindocarbocyanine perchlorate
AE: Adverse events
ATMP: Advanced therapy medicinal product
BM: Bone marrow
CCS: Canadian Cardiovascular Society
CFU-EC: Colony forming unit-endothelial cell
CMR: Cardiac magnetic resonance
CPET: Cardiopulmonary exercise testing
CT: Cell therapy
ECG: Electrocardiogram
EF: Ejection fraction
FBS: Fetal bovine serum
FLT3LG: Flt3 ligand
GMP: Good manufacturing practice
GRO-α: Growth-regulated oncogene alpha
HGF: Hepatocyte growth factor
ICD: Implantable cardioverter defibrillator
ICE: Intracardiac echocardiography
IL: Interleukin
LIF: Leukemia inhibitory factor
LV: Left ventricle
MACE: Major adverse cardiac events
MCP-1: Monocyte chemoattractant protein-1
MCS: Mental Component Summary
MI: Myocardial infarction
MIP-1β: Macrophage inflammatory protein-1 beta
MLHFQ: Minnesota Living with Heart Failure Questionnaire
MoA: Mode of action
NYHA: New York Heart Association
PCI: Percutaneous coronary intervention
PCS: Physical Component Summary
PDGF-bb: Platelet-derived growth factor type bb
RA: Refractory angina
RANTES: Regulated on activation normal T cell expressed and secreted
RCT: Randomized controlled trials
SAE: Serious adverse events
SCF: Stem cell factor
SDS: Summed difference score
SF-12: Short Form-12
SOP: Standard operative procedure
SPECT: Gated-single photon emission computed tomography
SRS: Summed rest score
SSS: Summed stress score
UAE-1: *Ulex europaeus* agglutinin-1
VEGF: Vascular endothelial growth factor
VO_2_: Oxygen consumption
WB: Washing buffer
WMSI: Wall motion score index
